# Effectiveness of educational outreach visits compared with usual guideline dissemination to improve family physician prescribing—an 18-month open cluster-randomized trial

**DOI:** 10.1186/s13012-018-0810-1

**Published:** 2018-09-05

**Authors:** Daniel Pinto, Bruno Heleno, David S. Rodrigues, Ana Luísa Papoila, Isabel Santos, Pedro A. Caetano

**Affiliations:** 1 Grupo de Investigação Académica Independente - Chronic Diseases Research Center, NOVA Medical School | Faculdade de Ciências Médicas, Universidade Nova de Lisboa, Lisbon, Portugal; 2 Department of Biostatistics and Informatics, NOVA Medical School | Faculdade de Ciências Médicas, Universidade Nova de Lisboa, Lisbon, Portugal

**Keywords:** Educational outreach, Academic detailing, Guideline adherence, Family practice, Drug utilization, Program evaluation, Cost-benefit analysis

## Abstract

**Background:**

Educational outreach visits are meant to improve the practice of health professionals by promoting face-to-face visits to deliver educational contents. They have been shown to change prescription behavior, but long-term effects are still uncertain. This trial aimed to determine if they improve family physician prescribing compared with passive guideline dissemination.

**Methods:**

Parallel, open, superiority, and cluster-randomized trial. National Health Service primary care practices (clusters) were recruited in the Lisbon region—Portugal between March 2013 and January 2014. They could enter if they had at least four family physicians willing to participate and not planning to retire in the follow-up period. Three national guidelines were chosen for dissemination: acid secretion modifiers, non-steroidal anti-inflammatory drugs, and antiplatelets. Physicians in the intervention group received one 15 to 20 min educational outreach visit at their workplace for each guideline. Physicians in the control group had access to guidelines through the Directorate-General for Health’s website (passive dissemination). Primary outcomes were the proportion of COX-2 inhibitors prescribed within the NSAID class and the proportion of omeprazole within the PPI class at 18 months after the intervention. A cost-benefit analysis was performed. Practices were randomized by minimization. Data analyses were done at individual physician level using generalized mixed-effects regression models. Participants could not be blinded.

**Results:**

Thirty-eight practices with 239 physicians were randomized (120 to intervention and 119 to control). Of 360 planned visits, 322 were delivered. No differences were found between physicians in the intervention and control groups regarding the proportion of omeprazole prescribed among PPIs 18 months after the visit (46.28 vs 47.15%, *p* = 0.971) or the proportion of COX-2 inhibitors among NSAIDs (12.07 vs 13.08%, *p* = 0.085). All secondary outcome comparisons showed no effect. There was no difference in cumulative drug costs at 18 months (3223.50€/1000 patients in the intervention group and 3143.92€/1000 patients in the control group, *p* = 0.848).

**Conclusions:**

Educational outreach visits were unsuccessful in improving compliance with guideline recommendations among Portuguese family physicians. No effects were observed at 1, 6, and 18 months after the intervention, and there were no associated cost savings.

**Trial registration:**

ClinicalTrials.gov NCT01984034. Registered 7 November 2013.

**Electronic supplementary material:**

The online version of this article (10.1186/s13012-018-0810-1) contains supplementary material, which is available to authorized users.

## Background

Clinical practice guidelines have the potential to improve the quality of care by summarizing current medical knowledge and promoting interventions with proven efficacy, safety, and cost-effectiveness [1]. Yet, issuing guidelines does not guarantee changes in clinical practice, as clinicians may not follow them for a variety of reasons. Among them are not being aware of or familiar with guidelines, considering they are ambiguous or disagreeing with recommendations, perceiving lack of self-efficacy, organizational constraints, and patient barriers [2, 3]. This may contribute to the problem of translating new medical knowledge to improvements in public health and of affordability of care [4, 5]. To address this problem, several guideline implementation strategies have been tried, with a systematic review finding small to moderate effects in a majority of trials [6].

Educational outreach visits are a type of strategy aimed at improving clinical practice [7, 8]. They consist of face-to-face visits done by an individual (henceforth named a detailer), usually a healthcare professional trained in communication skills, to physicians or other health workers in their own setting. During the visit, the detailer enquires about the physician’s baseline knowledge and motivations, presents content prepared by an independent organization with clear educational goals and using concise and graphic appealing materials, all the while stimulating physician interaction and providing positive reinforcement [9]. Visits are to one individual or a small group, in contrast with large educational meetings. A systematic review concluded that educational outreach visits have a small, but consistent and potentially important, effect on prescription improvement [8]. It also highlighted a gap in knowledge about the performance of this type of intervention in the long term (beyond 1 year). Trials using multifaceted interventions that included educational outreach to reduce inappropriate prescribing showed sustained effectiveness 1 year after the intervention [10–12], but it is unclear if educational outreach alone can achieve the same effect.

Portugal has a publicly funded National Health Service (NHS) with universal coverage providing the majority of primary care [13]. Primary care services are organized in small local practices, with 4 to 12 family physicians, plus a roughly equal number of nurses and a smaller number of secretaries [14]. In some practices, physicians are paid a fixed salary (personalized care units), while in others, they can have pay for performance incentives (family health units). Patients are registered with a single family physician, but may visit other physicians within the same practice if theirs is unavailable. Physicians in a practice meet frequently to discuss organization of care and performance indicators (which include items on prescribing). The Directorate-General for Health (an agency of the Ministry of Health) is responsible for issuing prescribing guidelines [15, 16]. Guidelines are made available on the agency’s website, and all health professionals are supposed to abide by them. This makes all physicians in Portugal exposed to the guidelines simultaneously. Guidelines are also available contextually on the Ministry of Health’s prescribing software and can be used as part of local clinical audits. However, their effectiveness in changing actual practice has not been studied.

In Portugal, family physicians work in group-based practices. Although educational outreach visits are directed at individual physicians, contamination among physicians working in the same practice is a plausible concern. In addition, a public health program of educational outreach visits would probably be delivered to all doctors in the same practice to minimize costs and loss of detailer time traveling. Thus, it may be more reasonable to assess educational outreach visits in the context of Portuguese primary care using a cluster-randomized design.

The primary objectives of the Trial to Assess the Effectiveness of Educational Outreach in Prescription Guidelines (TEP trial) [17] were to determine if educational outreach visits, compared with passive guideline dissemination, resulted in a reduction of the proportion of COX-2 inhibitors prescribed among the NSAID class and an increase in omeprazole prescriptions among the PPI class 18 months after the visit (long term). Secondary objectives were the effects on the same drugs at 1 (short term) and 6 months (medium term), and the short-, medium-, and long-term effects in the prescription of clopidogrel, thus the duration or persistence of effect post intervention. The trial also aimed to determine the cost-benefit of educational outreach visits.

## Methods

The protocol for this trial has been published previously, along with a PaT plot, and a cascade diagram [17].

### Trial design

The TEP trial aimed to determine the long-term effectiveness of educational outreach visits and their cost-benefit relation. It was a parallel, open, superiority, cluster-randomized controlled trial conducted in Portuguese primary care physicians. Clusters were Portuguese NHS primary care practices.

### Participants

The trial recruited family physicians working in NHS practices of the Lisbon region, Portugal, between March 2013 and January 2014. A practice would be eligible to participate if it had at least four physicians. All family physicians were eligible, except those planning to retire or without a stable patient list. Participants were recruited through practice coordinators. Family practices were the units of randomization. There was no financial incentive to participation. Participating physicians completed a baseline characteristic questionnaire and consented to schedule educational outreach visits as well as to the collection of their aggregate prescription data.

### Interventions

A detailed description of the intervention is provided in Additional file 1 as a TIDieR checklist [18].

Three guidelines were chosen for dissemination: non-steroidal anti-inflammatory drugs (NSAID), acid secretion modifiers, and proton pump inhibitors (PPI) and antiplatelets [19–21]. These guidelines had been issued by the Directorate-General for Health to address inappropriate prescribing and high spending in these drug classes. Physicians randomized to intervention clusters received three educational outreach visits during a 6-month period. Key messages were identified in each guideline. The NSAID guideline advocated for less use of COX-2 inhibitors, recommending they would only be prescribed to patients with increased gastrointestinal risk who did not tolerate a classical NSAID with a gastroprotective agent. For acid secretion modifiers, the guideline recommended that omeprazole should be preferred as it was as effective as other proton pump inhibitors and less expensive. The antiplatelets guideline recommended less use of clopidogrel, which should not be maintained long term after myocardial infarction, acute coronary syndrome, or percutaneous coronary intervention. Thus, we had a diverse mix of objectives aimed at improving rational prescribing: in one case to increase the usage of a specific drug and in two other cases to decrease drug usage.

Each visit was planned to focus on one guideline, last from 15 to 20 min and had one family physician present (up to three were allowed if physicians preferred, but one-to-one visits were encouraged). The visit would begin with an introduction about the detailer and the purpose of the visits, confirming the physician’s availability. Then educational needs would be assessed by asking about the physician’s usual practice with open questions. These would shape how the detailer would deliver key messages about the guideline, addressing scientific evidence, and benefits of following the guideline, barriers, and facilitators of change. The physician would be given the opportunity to present objections, which were addressed by the detailer. The visit ended with a summary and encouragement for the physician to commit to change. A point of care summary was distributed with each visit, and a brochure was used by the detailer as a visual aid. Copies of the brochures and point of care summaries are made available in Additional file 2.

Whenever possible, a single detailer performed all three visits to the same physician. Visits could take place in between patient visits or at other times indicated by the physician. Visits could be rescheduled up to the day before they were planned, but if the physician was unable to attend and could not warn the detailer beforehand, that visit would be skipped. Detailers filled a short questionnaire at the end of each visit, included those that were not made.

The detailers were three members of the research team (two family physicians and one academic pharmacologist) and nine physicians that were trained for the trial (six family physicians and three family medicine residents). All detailers were trained on the principles of educational outreach and the contents of each visit, to ensure consistency.

For the control group, usual guideline implementation consisted of passive dissemination by their publication on the Directorate-General for Health’s website.

### Outcomes

The trial had two primary outcomes, measured at the physician level: the proportion of COX-2 inhibitors prescribed within the NSAID class and the proportion of omeprazole within the PPI class, both measured in defined daily doses (DDD) at 18 months after the intervention. There were seven secondary outcomes: the same proportions of COX-2 inhibitors and omeprazole measured at 1 and 6 months after the intervention and the number of clopidogrel DDD per 1000 registered patients at 1, 6, and 18 months after the intervention.

We also conducted a cost-benefit analysis using the sum of all prescriptions dispensed for NSAIDs, acid suppressive therapy (PPIs, H2-receptor antagonists, misoprostol, and reimbursed anti-acids), and clopidogrel from month 1 following the intervention until month 18. Costs were considered from the perspective of a government-supported program, with government as the payer; therefore, only the reimbursed portion of drugs was considered. Differences in costs between the intervention and control group would be compared with the cost of training and paying detailers, preparing and printing educational materials, program coordination, and indirect costs of physician time (spent with a detailer rather than seeing a patient).

Only prescriptions that were dispensed were counted. Drug dispensing and cost data was provided by the Lisbon Regional Health Administration. In the Portuguese health system, prescriptions can be made for acute or chronic conditions. The former are valid for dispensing within 30 days and the later for 6 months. Of the studied drugs, only NSAIDs cannot be prescribed for chronic use. Physicians who transferred to other practices within the health region were followed, and their prescriptions monitored. When prescription data was not available, the last known month’s prescription was used.

### Sample size

Pilot data was obtained from three primary care practices and was used to estimate within unit variability and the intra-cluster correlation coefficient. Aggregate data from the Regional Health Administration was used to estimate the mean prescription and standard deviation for primary outcomes. Our sample size was calculated assuming the intervention would lead to a 5% absolute difference in compliance with guidelines between intervention and control units for primary outcomes, a mean cluster size of six physicians per practice, a 1:1 allocation ratio of controls per intervention unit, an alpha type error of 0.025, and a dropout rate of about 15%. To achieve 80% power, a sample of 110 physicians in each group was needed. To recruit the necessary 220 physicians, 38 primary care units would be required. STATA 12.0 (STATA Corp, TX, USA) and its sampsi and sampclus commands were used to calculate a sample size.

### Randomization

Clusters were allocated to the intervention or control groups using minimization, a method to achieve good balance regarding baseline characteristics that could influence the outcomes when the number of clusters is small [22]. We stratified for number of physicians in a practice, median baseline prescription of COX-2 inhibitors and omeprazole (above or below the regional median), proportion of physicians with fewer than 10 years of practice after completing vocational training, and type of primary care practice. The sequence of intervention visits for each practice was determined by simple randomization using Random.org sequence generator [23].

Allocation was concealed by having the project manager assign a sequential number to each practice as it completed enrollment. The trial statistician received only anonymized data (sequential number and minimization variables), blindly allocated practices to each trial arm, and returned allocation information to the trial manager.

Neither participating physicians nor detailers could be blinded. Outcomes were collected independently from the researchers by the Regional Health Administration and were only provided after the intervention had ended. Unlike what was planned in the protocol, the lead author could not be blinded to group and visit sequence allocation because the Regional Health Administration needed to consult with study author for data extraction. However, we were able to keep the trial statistician blinded.

### Statistical methods

Analysis was performed using the intention to treat principle. Physicians who transferred to another unit in the region were followed. For those we were unable to retrieve prescription data (transferred to another region or stopped working), we used the last working month’s prescription. Outcomes in both groups were compared using generalized mixed-effects multi-level regression models using the primary care practice to account for clustering. For model fitting, proportions of omeprazole and COX-2 inhibitors were logarithmically transformed [ln(*x*/(1 − *x*)] because of non-normal distributions. Intra-cluster correlation coefficients were calculated for primary outcomes. Statistical significance was assumed for a *p* value less than 0.025. STATA 12.0 (STATA Corp, TX, USA) was used to conduct the analysis. No interim analyses were done.

### Ethical approval

The trial was approved by the ethics committees of the Lisbon Regional Health Administration and NOVA Medical School. Family physicians invited to participate received written information about the main aspects of the trial, and participants gave consent for researchers to access their prescription data. The trial only collected aggregated and non-identifiable patient data.

Given the intervention-posed minimal risks to patients, no data monitoring committee was established and no stopping guidelines were defined.

## Results

### Participants

**Fig. 1:**
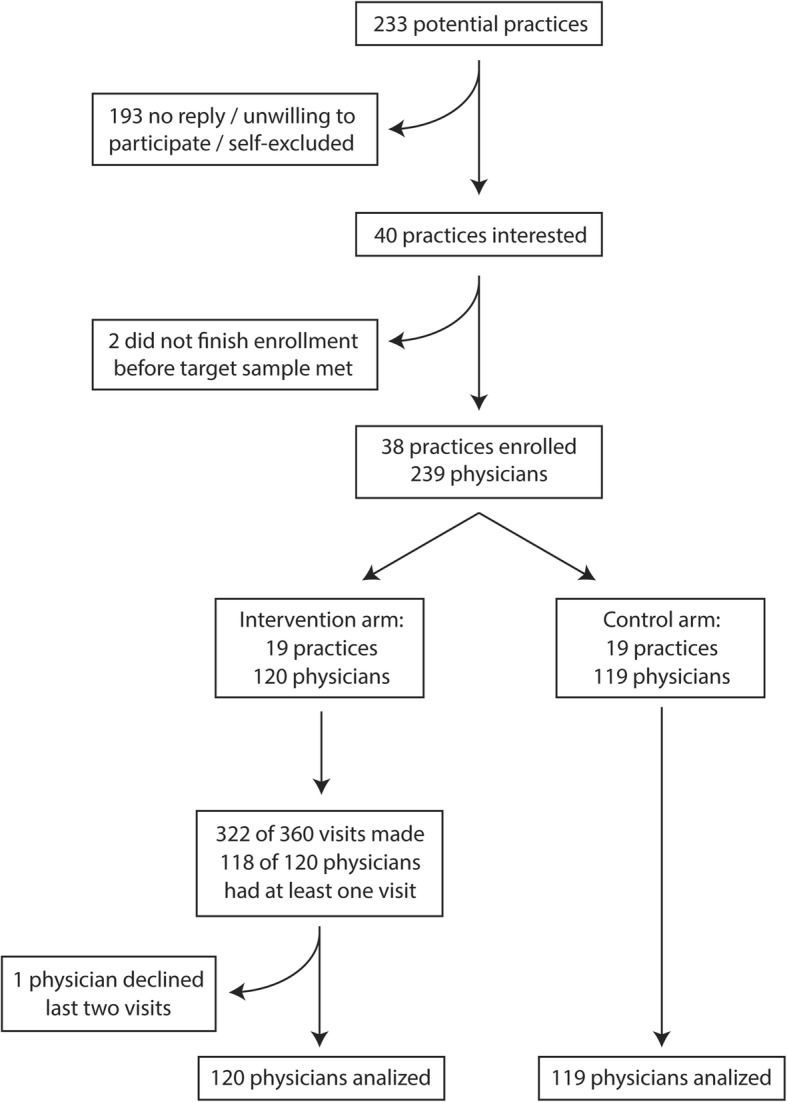
Participant flow diagram

Recruitment began in March 2013 and ended in January 2014. Participant flow chart is shown in Fig. 1. The research team met with practice coordinators from 13 of 15 health center groups in the Lisbon Region, representing 233 practices. Of these, 193 did not reply to subsequent contacts, were unwilling to participate, or self-excluded for having less than four physicians willing to participate. We randomized 38 clusters with 239 participating physicians.

Baseline characteristics for participants in the intervention and control groups are shown in Table 1. As expected, groups were balanced regarding the characteristics used for minimization. However, the number of patients per physician was higher for the intervention group.

**Table 1:** Baseline characteristics of participating physicians

	Intervention	Control
Physicians per cluster—mean (standard deviation)	6.3 (1.4)	6.3 (1.4)
Unit type—*n* units (*n* physicians)		
Family health unit	17 (107)	18 (114)
Personalized care unit	2 (13)	1 (5)
Female, %	70.8	79.0
Age—years, median (*P*_25_–*P*_75_)	52 (41–59)	47 (38–59)
Years in practice—median (*P*_25_–*P*_75_)	13 (6.5–28)	11 (4–28)
Residency tutor, %	55.8	52.9
Patient list size—median (*P*_25_–*P*_75_)	1874 (1812–1923)	1813 (1746–1872)
Prescription—mean (standard deviation)		
Omeprazole, %	47.13 (13.50)	48.30 (13.70)
COX-2 inhibitors, %	13.20 (9.69)	14.67 (12.88)
Clopidogrel, DDD/1000 patients	0.0986 (0.0502)	0.1053 (0.0491)
NHS expenditure with NSAIDs, acid secretion modifiers, and clopidogrel/month/1000—€, mean (standard deviation)	189.60 (73.41)	192.72 (85.67)

*P*_*25*_, first quartile, *P*_*75*_ third quartile, *COX-2* cycloxigenase-2, *DDD* defined daily dose, *NHS* National Health Service, *NSAIDs* non-steroidal anti-inflammatory drugs

### Delivery of the intervention

The intervention began in January and ended in June 2014. Of the 360 planned visits, 322 (89.4%) were done. Thirty visits failed because the physician was absent (21), unavailable (5), and on leave (2) or because the detailer was unavailable (2). Two physicians had none of the planned visits, as they were on extended leave. One physician was unavailable for the first visit and chose not to receive further visits. No physician withdrew consent to participate in the prescription analysis. Only one target physician was present in 89.1% of completed visits. The three visits were all made by the same detailer in 88.3% of physicians. Detailers reported delivering the full educational content in 97.8% of visits.

### Follow-up

Participating physicians were followed from January 2014 to December 2015. Prescription data was available for both primary outcomes in 96.7% of physicians in the intervention group; in the control group, it was available in 94.1% physicians for PPIs and 90.8% for NSAIDs. Secondary short-term outcomes (1 month after the intervention) were available for 98.3 and 99.2% of the control and intervention groups, respectively, and medium term (6 months after the intervention) for 96.9 and 97.6%. Overall, 29 physicians had one or more months without prescription data for one of the studied drugs (16 in the control group and 13 in the intervention group). Three physicians in the control group and two in the intervention group had no prescriptions of any of the studied drugs in the final 6 months of the study. For the remaining physicians, there were prescriptions after one or more months without data, suggesting temporary absences and not losses to follow-up.

### Intervention effects

**Fig. 2:**
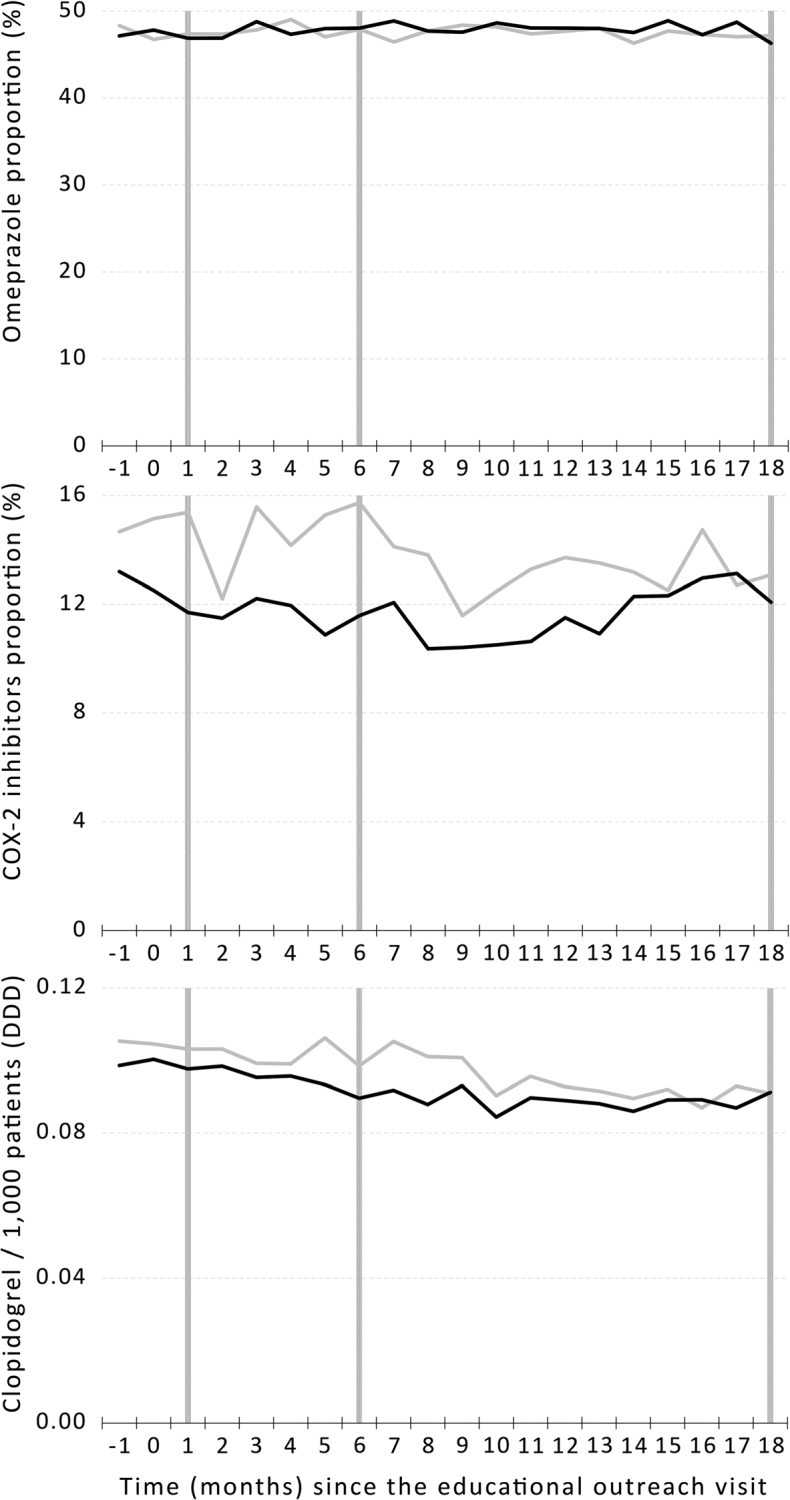
Prescription timeline of drugs in primary and secondary outcomes (vertical bars) from 1 month before the intervention (− 1) to 18 months after the intervention (18) in the intervention (black line) and control (gray line) groups

The results of the intervention are shown in Table 2 and Fig. 2. There were no differences between the intervention and control groups regarding primary outcomes: proportion of omeprazole among PPIs and proportion of COX-2 inhibitors among NSAIDs at 18 months after the intervention. The intra-cluster correlation coefficient was 0.305 (95% confidence interval 0.177–0.473) for omeprazole prescriptions at 18 months. No significant intra-cluster correlation existed for COX-2 inhibitors at 18 months. There were also no significant differences in secondary outcomes, in total costs or class-specific costs for the period between 1 and 18 months after the intervention.

**Table 2:** Prescription and cost of the studied drugs at 1, 6, and 18 months after the intervention

	Intervention (*n* = 120)	Control (*n* = 119)	*p*
Omeprazole, % (95%CI)^*^			
+ 1 month	46.86 (44.34–49.39)	47.36 (44.81–49.91)	0.744
+ 6 months	48.02 (45.58–50.46)	47.90 (45.01–50.79)	0.696
+ 18 months (primary outcome)	46.28 (43.77–48.79)	47.15 (44.39–49.91)	0.971
COX-2 inhibitors, % (95%CI)^¶^			
+ 1 month	11.70 (9.83–13.57)	15.38 (12.87–17.90)	0.131
+ 6 months	11.59 (9.28–13.89)	15.74 (13.42–18.05)	0.061
+ 18 months (primary outcome)	12.07 (9.75–14.41)	13.08 (10.75–15.41)	0.085
Clopidogrel, DDD/1000 (95%CI)			
+ 1 month	0.098 (0.886–0.107)	0.103 (0.094–0.112)	0.456
+ 6 months	0.090 (0.082–0.098)	0.099 (0.089–0.108)	0.230
+ 18 months	0.091 (0.083–0.100)	0.091 (0.082–0.100)	0.840
Cost 1–18 m/1000, € (95%CI)^¶^			
Gastric secretion modifiers	1647.79 (1541.37–1754.21)	1626.38 (1511.76–1741.01)	0.880
NSAIDs	1099.26 (984.70–1213.81)	983.02 (873.49–1092.55)	0.515
Clopidogrel	476.45 (428.34–524.57)	539.05 (491.22–586.87)	0.184
Total	3223.50 (2999.55–3447.44)	3143.92 (2917.61–3370.23)	0.848

*DDD* defined daily dose, *CI* confidence interval

*Intervention intended to increase this proportion

^¶^Intervention intended to decrease this proportion/absolute value

As there were no differences in costs between groups, we did not perform a formal cost-benefit analysis.

## Discussion

### Summary of main results

Our results showed no effect of educational outreach visits to align prescribing of family physicians with national guideline recommendations. Neither short-, medium-, nor long-term effects were seen, and no differences in drug costs were found between the intervention and control groups.

### Strengths and limitations

The planned intervention was adequately implemented: educational visits were delivered with a very high completion rate and were accepted by most participants, and the planned educational content was provided. The intervention was similar to what would have been a government-funded educational outreach program. Outcome data was collected from an administrative database, hence independently from the researchers and with few missing data points. We hypothesize that missing data points correspond to the absence from work due to illness, maternity or paternity leave, or other unforeseen causes and that it is likely that data is missing completely at random and not related with being assigned to the intervention or control groups. We could gather long-term follow-up data for most physicians, which allowed the study of the intervention’s effects over time. Only five among 239 physicians had no data points for outcomes in the last 6 months and may have been lost to follow-up (although we cannot be certain these were not temporary interruptions). Costs were considered for the whole pharmacological class and not just one drug (as costs could have been transferred between drugs within a class). Having the order of visits randomized between clusters allowed us to exclude effects of a possible build-up of the detailer-physician relation. Finally, results are consistent across the different drug classes studied.

The major limitation for the trial is that there was likely selection bias for participation. When compared with regional data, recruitment was higher among family health units than among personalized health care units, and the observed baseline proportions of COX-2 inhibitors and omeprazole were lower than in our pilot data for the region [17]. This suggests that physicians participating in trial had less margin for improvement. Our strategy may not have been effective because all physicians that volunteered to participate were already familiar with the guidelines (passive dissemination could have been enough), and we did not enable the intervention group to adequately address other existing barriers to implement the recommendations. Tools like patient handouts could have been provided to complement point of care summaries for physicians and help them change prescriptions. Other components could have been added to the intervention, such as audit and feedback on prescriptions. However, multifaceted interventions have not been shown to be superior to single-component interventions in changing health professionals’ behaviors [24, 25]. Our choice of drugs to be targeted by the intervention may have introduced influences from other specialists on family physicians’ prescriptions, limiting their ability to change a prescribed treatment [26]. Other drug classes, such as antibiotics, may be less susceptible to these influences, as prescriptions are one-off.

Although groups were balanced in most characteristics selected for minimization, there was some imbalance regarding patient list size. This certainly influenced absolute costs and is why we present costs per 1000 registered patients. It is uncertain if it could have influenced prescriptions, as baseline levels for the drugs of interest are similar. Another limitation was known from the outset and relates to prescription data. We used prescriptions that had been dispensed at a community pharmacy and not all prescriptions issued by the participating physicians. This was because information on issued prescriptions is less reliable in the database, and our pilot data showed a large percentage of prescribed drugs were never actually dispensed (due to factors like lack of patient adherence, mismatch between the patient’s needs and the number of prescribed packages, loss of prescriptions, and errors when issuing the prescription) [17]. Dispensing can occur up to 30 days after a prescription is issued for NSAIDs and up to 6 months for PPIs and clopidogrel. Hence, part of the prescriptions dispensed for PPIs and clopidogrel had not been issued in the same or the previous month.

Another issue to consider is the relatively large random monthly variation in prescription outcomes, which suggests our sample size may have been small to handle physicians with low prescription volume. Considering our choice of when to measure short-, medium-, and long-term outcomes is somewhat arbitrary, this may have been an issue when measuring short- and medium-term outcomes, where this fluctuation is more apparent for COX-2 inhibitors and clopidogrel.

### Interpretation of the results

A meta-analysis of educational outreach visits showed a median-adjusted risk difference in compliance with a desired prescribing practice of 4.8%, concluding educational outreach visits had an effect on prescribing, although heterogeneity was noted [8]. Since then, educational outreach visits have continued to yield heterogeneous results regarding effectiveness [27–31]. Educational outreach visits have also been integrated as part of multifaceted interventions, likewise with mixed results [10–12, 32–36].

Our study aimed to contribute to one of the unanswered questions in previous literature: if visit performance deteriorates in the long term. However, we were unable to find any short-, medium-, or long-term benefits of educational outreach visits. One possible reason for lack of effect is that physicians more interested in improving their prescribing behavior self-selected for the trial, and baseline prescription was already more compliant with guidelines than in physicians who did not volunteer to participate. It is possible that we did not target what Soumerai describes as “high-potential” physicians—those with prescribing patterns more distant from guideline recommendations; hence, more potential to change [9, 37]. Given the control group was also exposed to the same guidelines, it is also possible the intervention was not so effective when used as an add-on to existing dissemination strategies. However, passive dissemination of guidelines had been shown to be inferior to educational outreach visits and multifaceted interventions that included educational outreach [38, 39]. The Portuguese context may have been important regarding the effects of the intervention, as primary care physicians are government employed, their performance is monitored through quality and spending indicators, and the National Health Directorate’s position on guidelines is normative in nature [13].

Our intervention asked physicians to change a current behavior, promoting the increase in omeprazole use (instead of other proton pump inhibitors) and the decrease in use of COX-2 inhibitors (to be replaced by other NSAIDs) and clopidogrel (to be replaced by aspirin). Physicians would have to stop something they were already doing, in one case increasing and the other two decreasing prescriptions. De-adoption of low-value clinical practices may be different than adopting new activities, [40] and physicians may respond differently when asked to increase or decrease prescription of a given drug. Our trial did not show an effect on either type of prescription behavior.

Not having shown an effect in short- and medium-term outcomes limits our ability to conclude on the long-term effects of educational outreach visits. Even though there was no statistical significance, we could observe some separation between groups in NSAIDs between 2 and 5 months after the intervention and in clopidogrel between 5 and 8 months, both favoring the intervention group. A larger delay for clopidogrel is consistent with the fact that they are classified as chronic prescriptions which are valid for 6 months. However, even these small differences seem to disappear by the end of the follow-up period. This suggests that, even if we had found positive results in the short and medium term for the main outcomes, effects would deteriorate over time.

Costs throughout the follow-up period were not significantly reduced in the intervention group. Since the intervention was ineffective, the investment in producing educational materials, training detailers, and conducting the visits was inefficient.

### Implications for implementation science

Our results do not support the widespread adoption of educational outreach visits to change prescribing behavior. Contextual factors relating to the local setting, behavior being targeted, and type of program to be implemented should be considered and effectiveness evaluations should precede large scale programs.

## Conclusions

Educational outreach visits were unsuccessful in improving family physicians’ compliance with guideline recommendations to decrease the relative use of COX-2 inhibitors in NSAID prescriptions, increase the relative use of omeprazole in PPI prescriptions, and decrease clopidogrel use in antiplatelet prescriptions. No effects were observed at 1, 6, and 18 months after the intervention, and there were no associated cost savings. Contextual factors may have been important to this result and should be considered when introducing educational outreach programs. Data for a process evaluation of the trial was collected, and its analysis could help us understand why the intervention had no effect.

## Additional files

Additional file 1: TIDieR checklist. (DOCX 22 kb)

Additional file 2: Copies of the brochures and point of care summaries. (PDF 3619 kb)

## Abbreviations

CI: Confidence interval
DDD: Defined daily dose
NHS: National Health Service
NSAID: Non-steroidal anti-inflammatory drug
PPI: Proton pump inhibitors
