# Supplementary material for: Effectiveness of educational outreach visits compared with usual guideline dissemination to improve family physician prescribing—an 18-month open cluster-randomized trial
Source: Implement Sci. 2018 Sep 5;13:120. doi: 10.1186/s13012-018-0810-1 (PMC6126017; doi:10.1186/s13012-018-0810-1)
Supplement: Supplementary file 1 — TIDieR checklist. (DOCX 22 kb) [file 13012_2018_810_MOESM1_ESM.docx]

Template for Intervention Description and Replication (TIDieR) checklist^[[1]](#endnote-1)^

Pinto D, Heleno B, Rodrigues DS, Papoila AL, Santos I, Caetano PA. Effectiveness of educational outreach visits compared with usual guideline dissemination to improve family physician prescribing – an 18-month open cluster randomized trial.

# Brief name

## 1. Provide the name or a phrase that describes the intervention

Educational outreach visits to improve family physician prescribing.

# Why

## 2. Describe any rationale, theory, or goal of the elements essential to the intervention

Clinical practice guidelines have the potential to change clinical practice, but clinicians may not follow them.^[[2]](#endnote-2)^ Guideline implementation interventions have the potential to improve guideline adoption, hence, the quality of care provided to patients. Educational outreach visits are one such strategy.^[[3]](#endnote-3)^

The Portuguese National Health Directorate issues guidelines meant to improve quality of care. However, it is not known if they are actually effective in changing clinical practice.

# What

## 3. Materials: Describe any physical or informational materials used in the intervention, including those provided to participants or used in intervention delivery or in training of intervention providers. Provide information on where the materials can be accessed (such as online appendix, URL)

During their encounters with participating family physicians, detailers used a brochure as a visual aid to highlight each guideline’s key-messages. At the end of the visit, a point of care summary handout was given to each physician. Brochures and point of care summaries are provided (in Portuguese) in appendix 1. These materials were developed by the study authors using the guidelines being presented as source materials.^[[4]](#endnote-4),^^[[5]](#endnote-5)-^^[[6]](#endnote-6)^ Brochures and point of care summaries were used to train detailers and their comments helped improve the draft versions and arrive at the final materials used in the trial.

## 4. Procedures: Describe each of the procedures, activities, and/or processes used in the intervention, including any enabling or support activities

At recruitment, physicians were asked to state their preference regarding visiting schedules. Participating physicians were contacted by email or mobile phone to schedule the first visit. The second and third visits were agreed on at the end of the previous visit.

Detailers were asked to send reminders to physicians two days before each visit. Physicians could request rescheduling for a visit up to the day before it was to take place. If the physician was unavailable but did not request the visit to be rescheduled, that visit would be skipped. Visits would be discontinued if physicians changed their workplace or requested not to be visited again.

At the end of each visit, detailers were required to fill a post-visit questionnaire (even if the visit hadn’t been accomplished). These questionnaires allowed the trial steering committee to observe visit progress (and contact detailers if problems were noticed) and were used to gather details about the visit and the detailer’s perception of physician engagement and difficulties delivering contents. These questionnaires were also meant to provide data for a process evaluation of the trial.

Detailers managed their visiting schedule and entered post-visit questionnaires in a web-based platform built by the authors specifically for the trial.

# Who provided

## 5. For each category of intervention provider (such as psychologist, nursing assistant), describe their expertise, background, and any specific training given

The detailing team consisted of three of the authors (two trained as family physicians and one as a pharmacist, all faculty at NOVA Medical School), six family physicians, and three family medicine residents in their fourth and final year of training.

The three authors that acted as detailers had received training from the National Resource Center for Academic Detailing (Boston, USA). All other detailers received three group training sessions (12 hours total) prior to the trial and were requested to study at home before each session. Training was provided by the authors and contents included the theory and components of educational outreach visits, mock visits using the guidelines and materials prepared for the trial, videos of recorded mock visits, discussion sessions with guideline authors and researchers and an online support group. Competency was assessed by the authors during training sessions.

# How

## 6. Describe the modes of delivery (such as face to face or by some other mechanism, such as internet or telephone) of the intervention and whether it was provided individually or in a group

The intervention was delivered in face to face visits. One detailer to one physician visits were encouraged. If physicians requested, up to three of them could be present in the same visit. Residents and medical students accompanying the physician could also be present.

# Where

## 7. Describe the type(s) of location(s) where the intervention occurred, including any necessary infrastructure or relevant features

Visits occurred in the physician’s practice – in their own office, another physician’s office or the practice’s meeting room.

# When and How Much

## 8. Describe the number of times the intervention was delivered and over what period of time including the number of sessions, their schedule, and their duration, intensity, or dose

Each physician was visited three times, one per guideline. Visits were planned to last 15 to 20 minutes. One or two visits could be done each month, depending on detailer and physicians’ availability. All visits referring to a guideline within a practice had to be delivered in the same month for all physicians.

Visits could be scheduled before, after or in between patient appointments, depending on physician preference and detailer availability.

# Tailoring

## 9. If the intervention was planned to be personalized, titrated or adapted, then describe what, why, when, and how

During each visit, detailers assessed the physician’s educational needs and used these to tailor the delivery of educational contents. Tailoring consisted mainly of emphasizing areas where the physician was less knowledgeable and spending less time discussing issues where the physician demonstrated being proficient.

# Modifications

## 10. If the intervention was modified during the course of the study, describe the changes (what, why, when, and how)

No modifications were made to the intervention during the trial by the study authors. Yet, nine minor protocol deviations were recorded: seven concerning changes in the planned order for the visits, two regarding detailer unavailability to perform visits and one by delivering a visit to four physicians at the same time.

# How well

## 11. Planned: If intervention adherence or fidelity was assessed, describe how and by whom, and if any strategies were used to maintain or improve fidelity, describe them

Adherence and fidelity were assessed during the trial using post-visit questionnaires to monitor detailer progress and delivery of educational contents. When deviations were noted, detailers were contacted by the trial steering committee to correct them.

## 12. Actual: If intervention adherence or fidelity was assessed, describe the extent to which the intervention was delivered as planned

The overall visit success rate was 89.4%, with 322 visits accomplished out of 360 planned. The full educational content was delivered in 97.8% of visits. In the remaining, the most common reason reported by the detailers for not delivering the full content was physician lack of time. All planned stages of the visit were followed by detailers in 89.4% of visits.

# References

1. Hoffmann TC, Glasziou PP, Boutron I, Milne R, Perera R, Moher D, et al. Better reporting of interventions: template for intervention description and replication (TIDieR) checklist and guide. BMJ. 2014 Mar 7;348(mar07 3):g1687–g1687. [↑](#endnote-ref-1)
2. Cabana MDRC: Why don’t physicians follow clinical practice guidelines? A framework for improvement. JAMA 1999, 282:1458–1465. [↑](#endnote-ref-2)
3. Soumerai SB. Principles of Educational Outreach ('Academic Detailing’) to Improve Clinical Decision Making. JAMA: The Journal of the American Medical Association. 1990 Jan 26;263(4):549. [↑](#endnote-ref-3)
4. Heleno B, Caetano PA, Pinto D, Monteiro E, Santos I: Norma 013/2011: Anti-inflamatórios não esteróides sistémicos em adultos: orientações para a utilização de inibidores da COX-2 [Internet]. Lisbon, Direcção-Geral da Saúde; 2013-02-13. [cited 2017-09-26]. Available from: https://www.dgs.pt/directrizes-da-dgs/normas-e-circulares-normativas/norma-n-0132011-de-27062011-atualizada-a-13022013-jpg.aspx [↑](#endnote-ref-4)
5. Caetano PA, Heleno B, Pinto D, Monteiro E, Santos I: Norma 036/2011: Supressão Ácida: Utilização dos Inibidores da Bomba de Protões e das suas Alternativas Terapêuticas [Internet]. Lisbon, Direcção-Geral da Saúde; 2011-09-30. [cited 2017-09-26]. Available from: https://www.dgs.pt/directrizes-da-dgs/normas-e-circulares-normativas/norma-n-0362011-de-30092011-jpg.aspx. [↑](#endnote-ref-5)
6. Pinto D, Caetano PA, Heleno B, Monteiro E, Santos I: Norma 014/2011: Utilização e seleção de Antiagregantes Plaquetários em Doenças Cardiovasculares [Internet]. Lisbon, Direcção-Geral da Saúde; 2013-07-08. [cited 2017-09-26]. Available from: https://www.dgs.pt/directrizes-da-dgs/normas-e-circulares-normativas/norma-n-0142011-de-14072011-atualizada-a-08072013-jpg.aspx. [↑](#endnote-ref-6)
