# Supplementary material for: Effectiveness of educational outreach visits compared with usual guideline dissemination to improve family physician prescribing—an 18-month open cluster-randomized trial
Source: Implement Sci. 2018 Sep 5;13:120. doi: 10.1186/s13012-018-0810-1 (PMC6126017; doi:10.1186/s13012-018-0810-1)
Supplement: Supplementary file 2 — Copies of the brochures and point of care summaries. (PDF 3619 kb) [file 13012_2018_810_MOESM2_ESM.pdf]

Grupo de Informação Académica Independente
Faculdade de Ciências Médicas - Centro de Estudos de Doenças Crónicas (CEDOC)

Anti-inflamatórios não esteroides

Como se comparam os AINE em termos de eficácia?

Número necessário tratar para obter pelo menos 50% de alívio durante 4 a 6 horas em pessoas com dor moderada a grave.

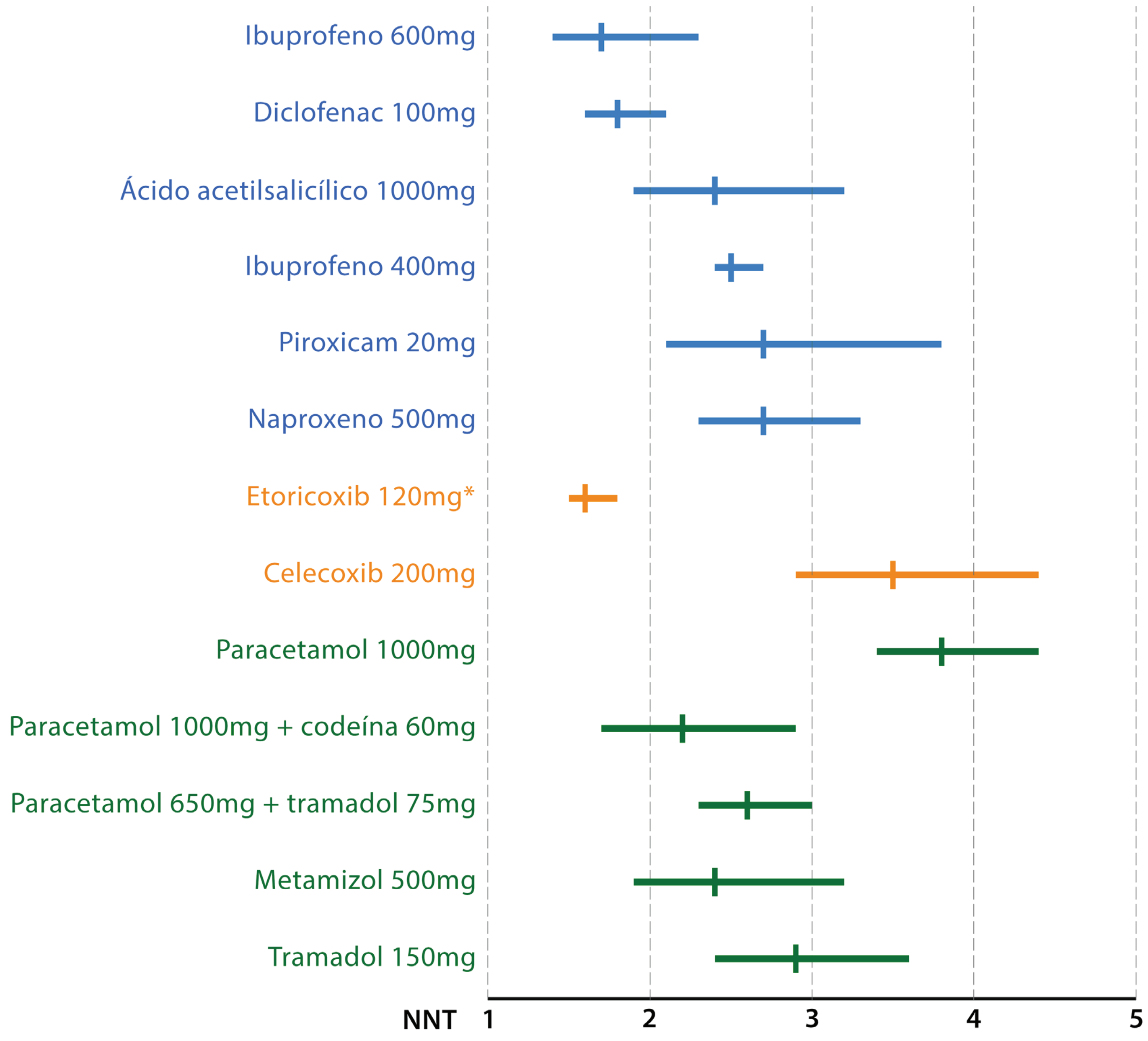

* A dose de etoricoxib aprovada para tratamento da osteoartrose é de 30 a 60mg por dia.

Fonte: The 2007 Oxford league table of analgesic efficacy. Bandolier. Disponível em: http://www.medicine.ox.ac.uk/bandolier/booth/painpag/Acutrev/Analgesics/Acutepain2007.pdf [acedido a 29/04/2013]

Financiado por Ministério da Saúde / INSA, I.P

E em termos de segurança?

Cardiovascular

Risco de eventos cardiovasculares major em comparação com o placebo.

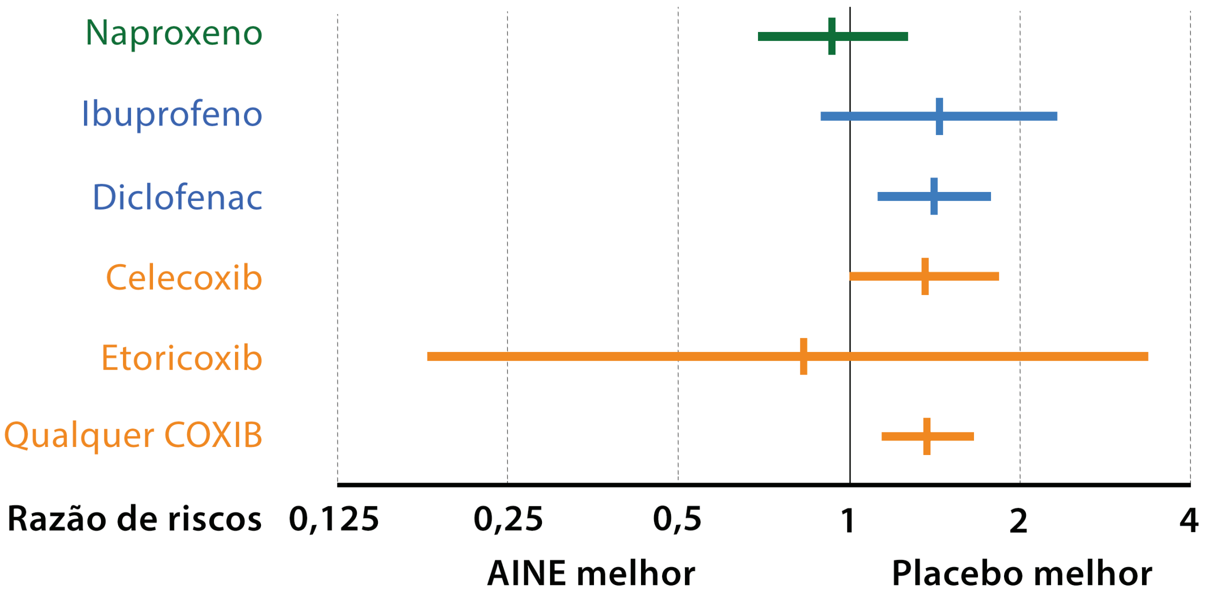

Gastrointestinal

Risco relativo de complicações gastrointestinais superiores face ao placebo.

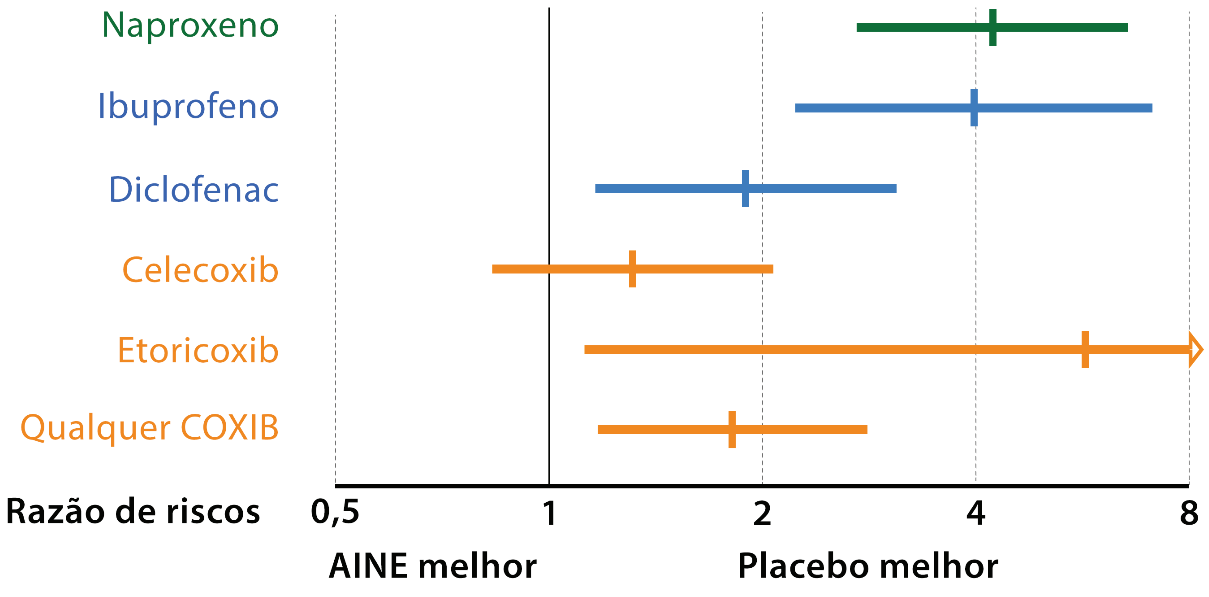

Naproxeno 1000mg/d
Ibuprofeno 2400mg/d
Diclofenac 150mg/d
Celecoxib 400mg/d
Etoricoxib 60-90mg/d

Fonte: Coxib and traditional NSAID Trialists' (CNT) Collaboration. Vascular and upper gastrointestinal effects of non-steroidal anti-inflammatory drugs: meta-analyses of individual participant data from randomised trials. Lancet. 2013 May 29. pii: S0140-6736(13)60900-9.

Inibidor da COX-2 ou AINE tradicional + IBP?

Probabilidade cumulativa de recorrência de hemorragia por úlcera.

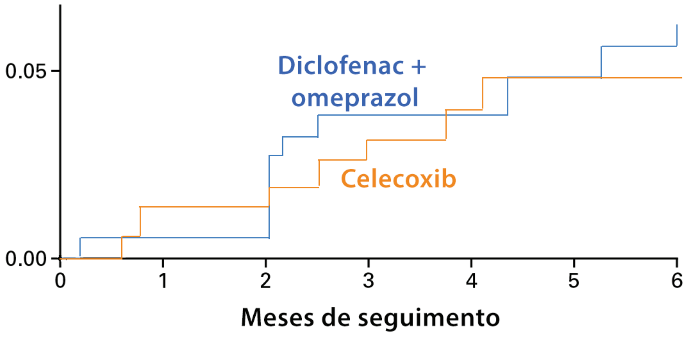

Fonte: Chan FK, Hung LC, Suen BY, et al. Celecoxib versus diclofenac and omeprazole in reducing the risk of recurrent ulcer bleeding in patients with arthritis. N Engl J Med. 2002 Dec 26;347(26):2104-10.

Quais os custos?

Custo mensal de tratamento para doses habituais de anti-inflamatórios.

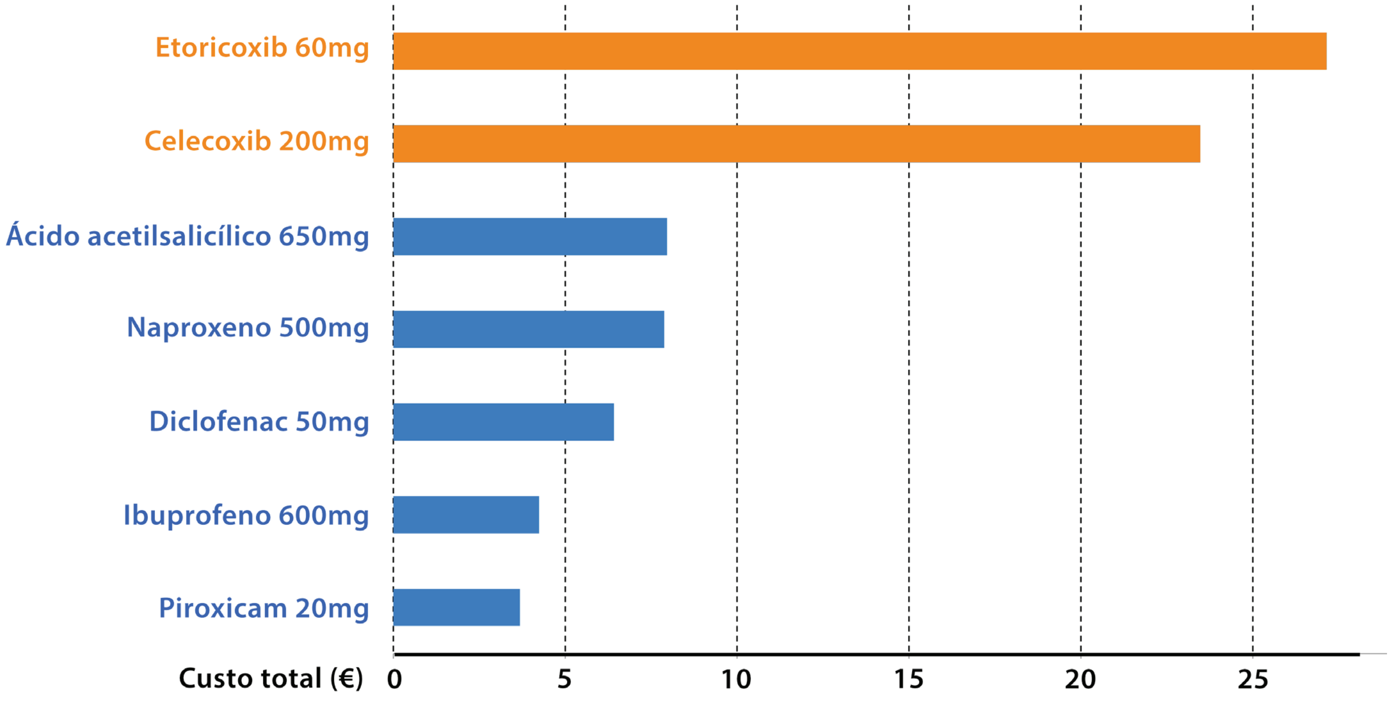

Fonte: Infarmed (1 de Maio de 2013)

O que diz a norma?

		Risco cardiovascular	
		Baixo	Alto
Risco gastrointestinal	Baixo	AINE clássico	Naproxeno
	Alto / sintomas	1ª opção: AINE clássico + gastroprotecção<br>2ª opção: inibidor COX-2	Evitar AINE

Fatores de alto risco para hemorragia gastrointestinal:

- doentes idosos (≥ 75 anos);
- antecedentes pessoais de úlcera péptica;
- utilização de corticosteroides sistémicos;
- utilização de anticoagulantes (varfarina ou outros)
- utilização concomitante de ácido acetilsalicílico
- infecção por Helicobacter pylori.

Fatores de alto risco cardiovascular:

- antecedentes pessoais de acidente vascular cerebral;
- antecedentes pessoais de acidente isquémico transitório;
- antecedentes pessoais de síndroma coronária aguda;
- angina estável;
- antecedentes pessoais de revascularização arterial;
- doença arterial periférica.

Medicamentos para gastroprotecção

- Inibidor da bomba de protões
- Antagonista dos receptores H_2 2x/dia
- Misoprostol

Anti-inflamatórios não esteróides

		Risco cardiovascular	
		Baixo	Alto
Risco gastrointestinal	Baixo	AINE clássico	Naproxeno
	Alto ou sintomas	1ª opção: AINE clássico + gastroprotecção<br>2ª opção: inibidor COX-2	Evitar AINE

Factores de alto risco para hemorragia gastrointestinal:

- doentes idosos (≥ 75 anos);
- antecedentes pessoais de úlcera péptica;
- utilização de corticosteroides sistémicos;
- utilização de anticoagulantes (varfarina ou outros)
- utilização concomitante de ácido acetilsalicílico
- infecção por Helicobacter pylori.

Factores de alto risco cardiovascular:

- antecedentes pessoais de acidente vascular cerebral;
- antecedentes pessoais de acidente isquémico transitório;
- antecedentes pessoais de síndroma coronária aguda;
- angina estável;
- antecedentes pessoais de revascularização arterial;
- doença arterial periférica.

Medicamentos para gastroprotecção:

- Inibidor da bomba de protões
- Antagonista dos receptores H_2 2x/dia
- Misoprostol

Pontos a reter

Não existem diferenças significativas entre os AINE quanto à eficácia analgésica.

Todos os AINE excepto o naproxeno se associam a aumento do risco de complicações cardiovasculares.

Nas pessoas com alto risco cardiovascular o anti-inflamatório preferencial deverá ser o naproxeno.

Todos os AINE aumentam o risco de complicações gastrointestinais, mesmo os inibidores da COX-2.

Nas pessoas com alto risco gastrointestinal ou com sintomas gastrointestinais de novo deve ser prescrita gastroproteção.

Os inibidores da COX-2 devem ser reservados para pessoas em risco acrescido de complicações gastrointestinais e que não toleram a associação AINE clássico + gastroprotecção.

Grupo de Informação Académica Independente
Faculdade de Ciências Médicas
Centro de Estudos de Doenças Crónicas (CEDOC)

Financiado por Ministério da Saúde / INSA, I.P

Grupo de Informação Académica Independente

Faculdade de Ciências Médicas - Centro de Estudos de Doenças Crónicas (CEDOC)

Modificadores da Secreção Ácida

Diagnóstico da pessoa com sintomas dispépticos

Sugestivo de DRGE*

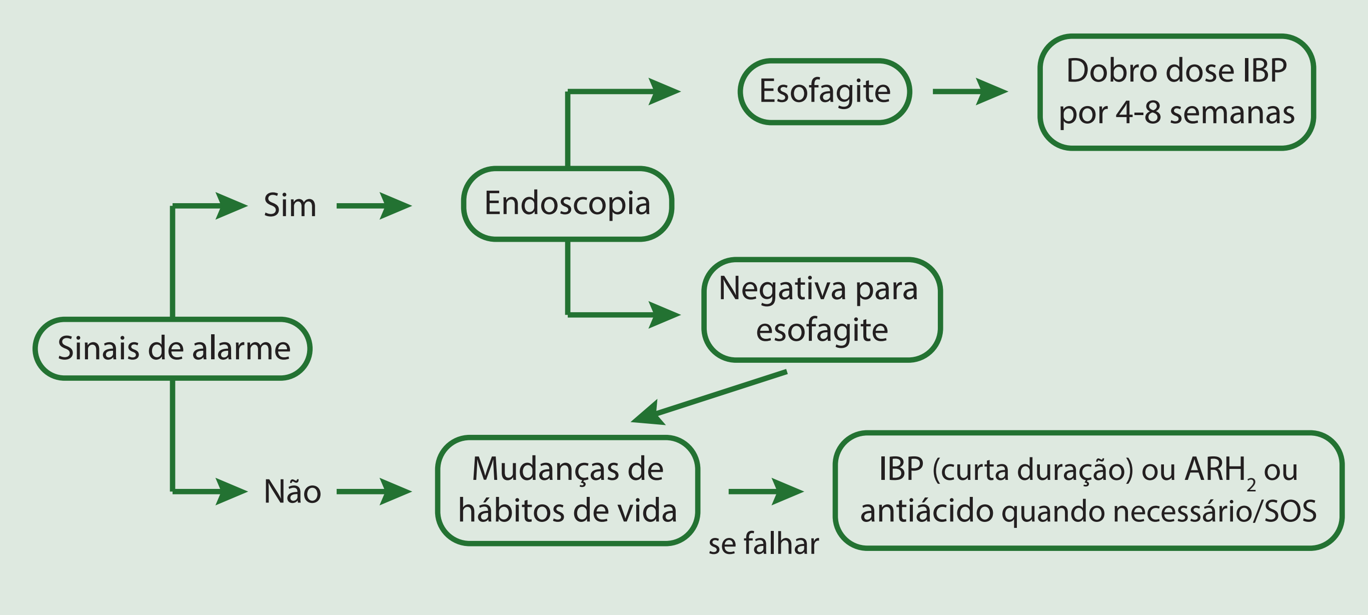

Não sugestivo de DRGE

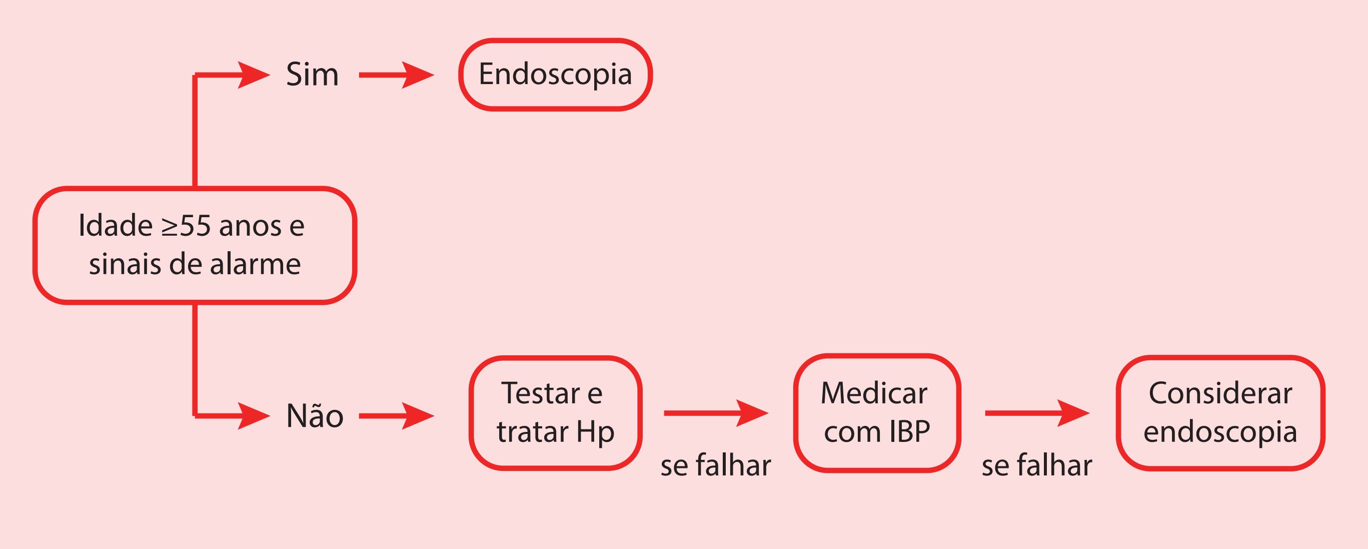

* Azia e/ou refluxo ácido como sintomas predominantes (> 1 episódio por semana).

Financiado por Ministério da Saúde / INSA, I.P

Que duração deve ter o tratamento com IBP?

Curto (4 a 8 semanas)

- Doença de refluxo gastro-esofágico sem esofagite (ou sem endoscopia)
- Úlcera péptica

Crónico

- Doença de refluxo gastro-esofágico com esofagite
- Dispepsia funcional (sem úlcera), se houver resposta
- Esófago de Barret
- Síndroma de Zollinger-Ellison
- Algumas pessoas medicadas com AINEs

O que utilizar em sintomas pontuais?

Tempo necessário para alívio dos sintomas

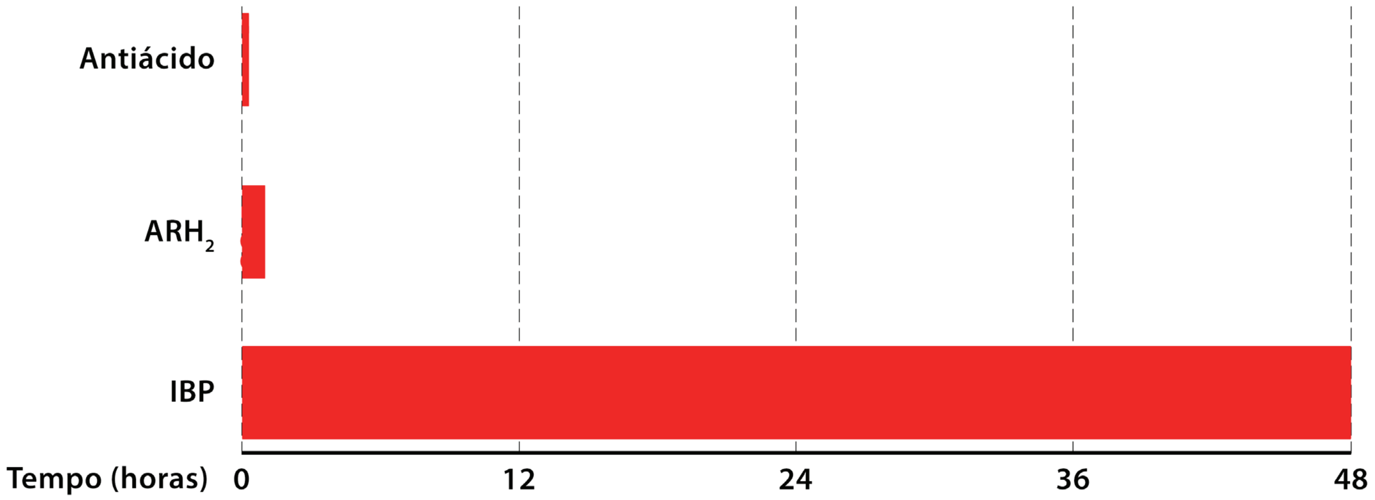

Por demorarem no início de acção, os IBP não são a melhor escolha para alívio rápido de sintomas pontuais.

Como se comparam os IBP em termos de eficácia?

Eficácia comparada dos IBP na cura da esofagite

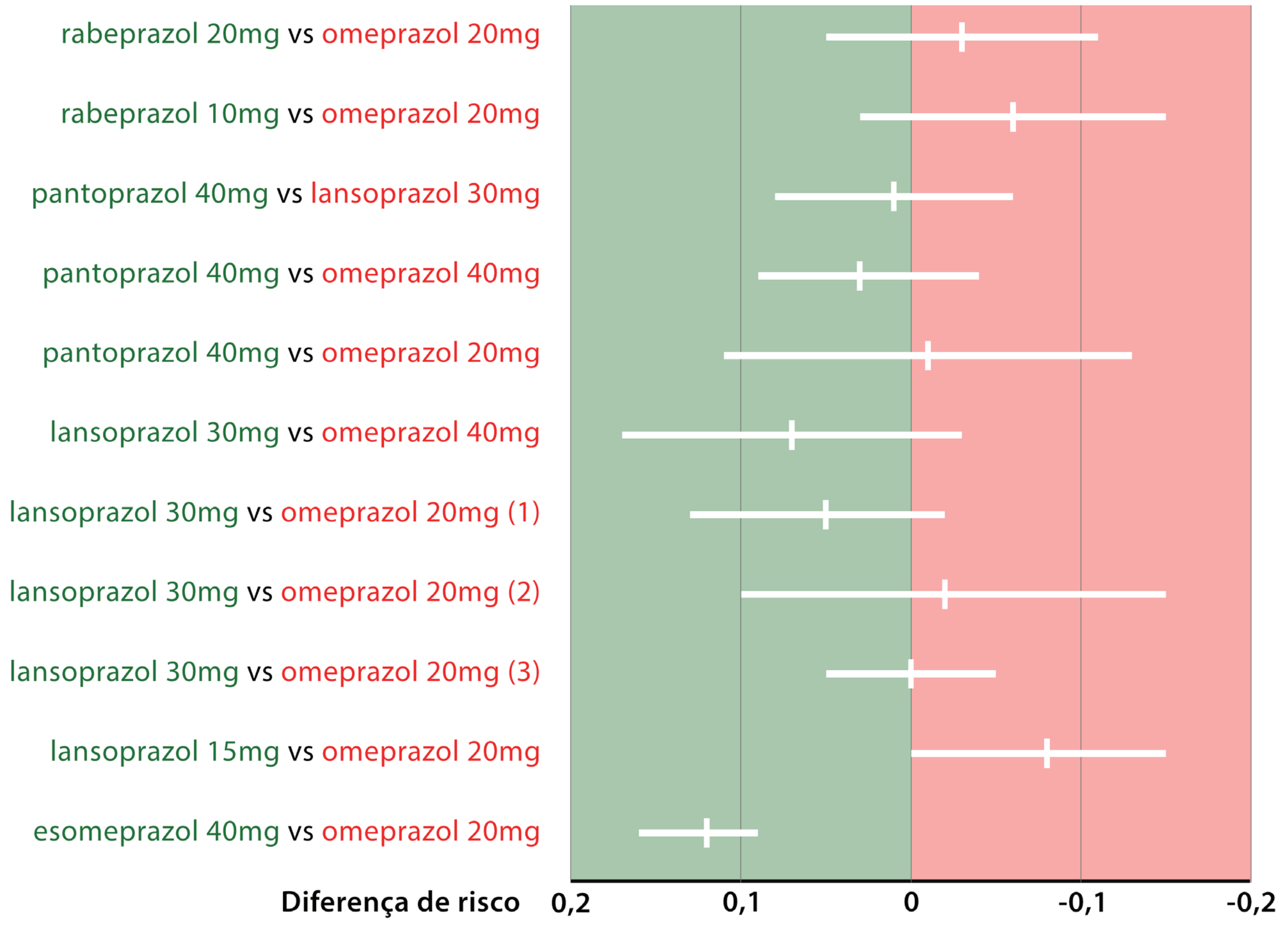

Qual o custo de tratamento?

Custo mensal de tratamento para doses habituais de modificadores da secreção ácida.

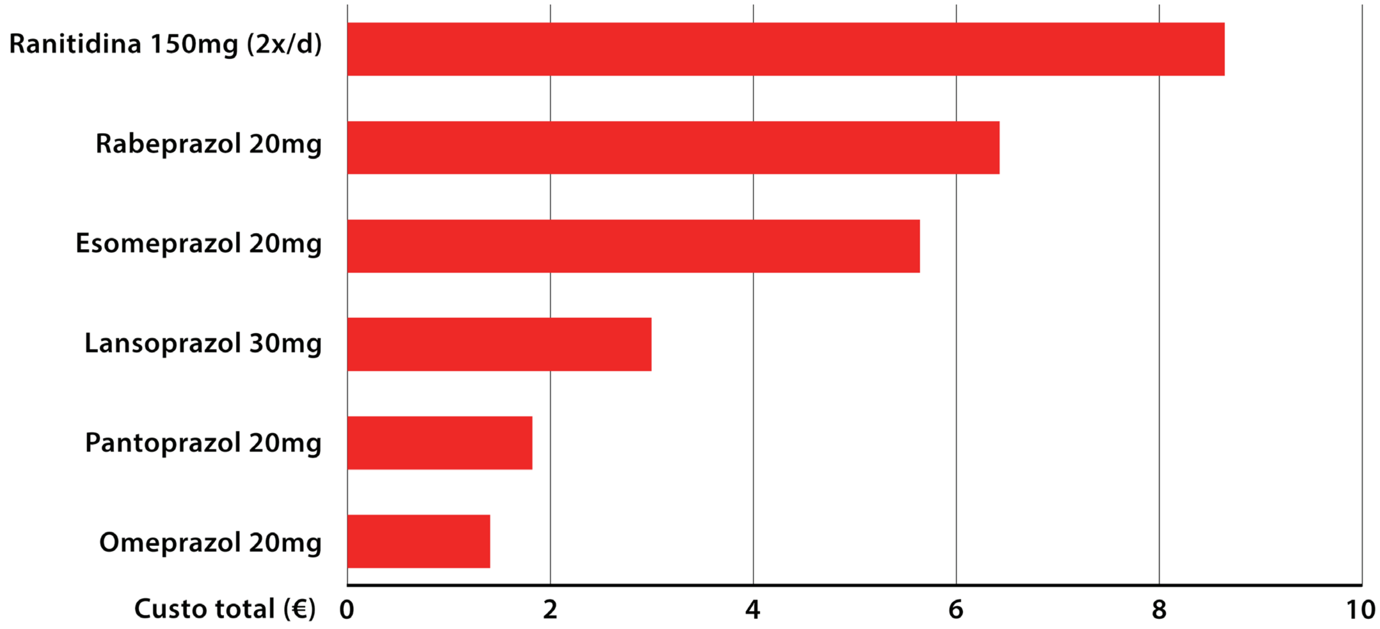

Fonte: Infarmed (1 de Maio de 2013)

Como interromper um IBP?

Doente com IBP
ex. omeprazol 20 mg por dia

→

Reduzir dose para metade durante 2 semanas
ex. omeprazol 20 mg em dias alternados

→

Parar IBP
Se necessário usar: antagonista dos receptores H_2 (ARH_2), ex. ranitidina, ou antiácido

→

Longo prazo
Reduzir gradualmente a dose de ARH2 ou antiácido para a dose mais baixa necessária para controlar os sintomas

Mudanças de estilos de vida na DRGE

- Perda de peso nas pessoas com excesso de peso ou obesidade;
- Elevação da cabeceira da cama;
- Evitar grandes refeições 2 a 3 horas antes de deitar;
- Evitar reclinar ou deitar nas 2 a 3 horas após uma refeição;
- Evitar os alimentos que o doente associa a agravamento dos sintomas;
- Pouca evidência para recomendar evicção de alimentos se não existir relação com sintomas.

Esquema sugerido para erradicação do H pylori

Terapia tripla durante 10 a 14 dias:

- IBP - Dose normal, duas vezes por dia, e
- Amoxicilina – 1 g, duas vezes por dia
  ou metronidazol – 500mg, duas vezes por dia – nos doentes com história de alergia
- Claritromicina – 500 mg, duas vezes por dia.

Modificadores da Secreção Ácida

Sinais de alarme para realização de endoscopia digestiva alta - dispepsia associada a:

- disfagia
- hematemese
- hemorragia gastrointestinal
- modificações do trânsito intestinal
- anemia
- odinofagia
- história anterior de neoplasia ou úlcera gastrointestinal
- vómitos recorrentes
- anorexia
- perda inexplicável de peso
- enfartamento
- massa abdominal
- hepatomegalia
- linfadenopatias

Não explicados por outros dados da história clínica.

Erradicação do H pylori

- IBP - dose normal, 2x por dia
- Amoxicilina - 1g, 2x por dia
  (metronidazol 500mg, 2x por dia nos doentes alérgicos)
- Claritromicina - 500mg, 2x por dia

10 a 14 dias

Pontos a reter

O tratamento dos sintomas dispépticos depende da indicação.

Tratamento curto (4-8 semanas)

- DRGE sem esofagite
- Úlcera péptica

Tratamento crónico

- DRGE com esofagite
- DRGE sem esofagite, mas com sintomas persistentes
- Esófago de Barret
- Úlcera péptica com episódios recorrentes
- Síndroma de Zollinger-Ellison
- Algumas pessoas medicadas com AINE
- Dispepsia funcional (se houver resposta)

Não existem diferenças significativas quanto à eficácia dos IBP.

O uso prolongado de IBP associa-se a efeitos adversos.

Alguns doentes poderão ter hipersecreção rebound, considere reduzir gradualmente a dose em vez de interrupção abrupta nas pessoas em tratamento prolongado.

Os IBP demoram 24 a 48 horas a aliviar os sintomas, pelo que não são a melhor escolha para alívio de sintomas pontuais.

Grupo de Informação Académica Independente
Faculdade de Ciências Médicas
Centro de Estudos de Doenças Crónicas (CEDOC)

Financiado por Ministério da Saúde / INSA, I.P

Antiagregantes plaquetários

Que antiagregante escolher?

Indicação terapêutica	Tratamento recomendado
Síndroma coronária aguda	Clopidogrel + Ácido acetilsalicílico até 1 ano<br>Ácido acetilsalicílico depois
Intervenção coronária percutânea	Clopidogrel + Ácido acetilsalicílico até 1 ano<br>Ácido acetilsalicílico depois
Enfarte do miocárdio há +1 ano	Ácido acetilsalicílico<br>Clopidogrel pode ser alternativa em alguns doentes
Angina estável	Ácido acetilsalicílico
AVC isquémico / AIT	Ácido acetilsalicílico ou Clopidogrel*<br>ou Ácido acetilsalicílico + dipiridamol
Doença arterial periférica	Ácido acetilsalicílico ou Clopidogrel

* O clopidogrel foi estudado em pessoas com antecedentes de AIT, mas não tem indicação aprovada nesse grupo.

Prevenção primária

Ponderar riscos e benefícios, não recomendado em geral. Só o ácido acetilsalicílico foi estudado.

Triflusal

Menor evidência e mais caro que o ácido acetilsalicílico.

Ticlopidina

Maior risco de efeitos adversso e menos estudado que o clopidogrel.

Financiado por Ministério da Saúde / INSA, I.P

Quando usar antiagregação dupla?

Pessoas com enfarte recente ou angina instável

Ácido acetilsalicílico vs clopidogrel + ácido acetilsalicílico no ensaio CURE (morte por doença cardiovascular, enfarte do miocárdio não fatal ou AVC).

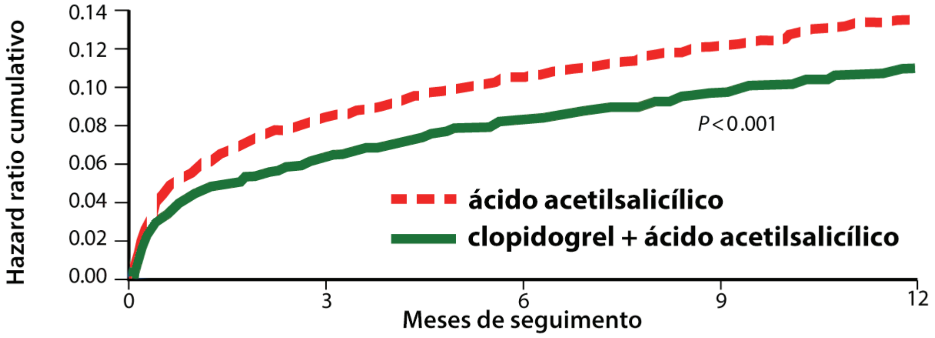

Pessoas com enfarte antigo ou angina estável

Ácido acetilsalicílico vs clopidogrel + ácido acetilsalicílico no ensaio CHARISMA (morte por doença cardiovascular, enfarte do miocárdio não fatal ou AVC).

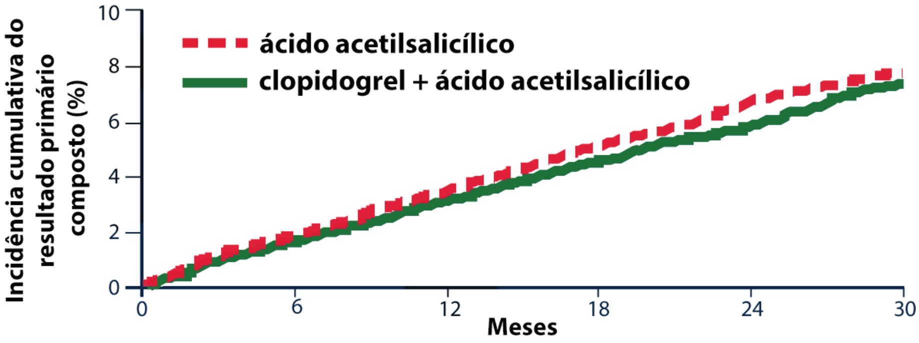

Subgrupos que potencialmente beneficiam do clopidogrel (incerto)

- Doença coronária ou
- Acidente vascular cerebral ou
- Acidente isquémico transitório

\+

- Cirurgia de bypass
- Episódios em múltiplos territórios
- ≥2 episódios isquémicos
- Diabetes

Benefício hipotético, existe pouca evidência científica para suportar esta opção.

Qual o tratamento mais eficaz após um AVC?

Terapia dupla com	Eficácia	Hemorragia	Comparada com:
Ácido acetilsalicílico + clopidogrel	Igual a	Mais que	Ácido acetilsalicílico
	Igual a	Mais que	Clopidogrel
Ácido acetilsalicílico + dipiridamol	Melhor que	Igual a	Ácido acetilsalicílico
	Igual a	Mais que	Clopidogrel

E na doença arterial periférica?

Clopidogrel vs ácido acetilsalicílico no estudo CAPRIE

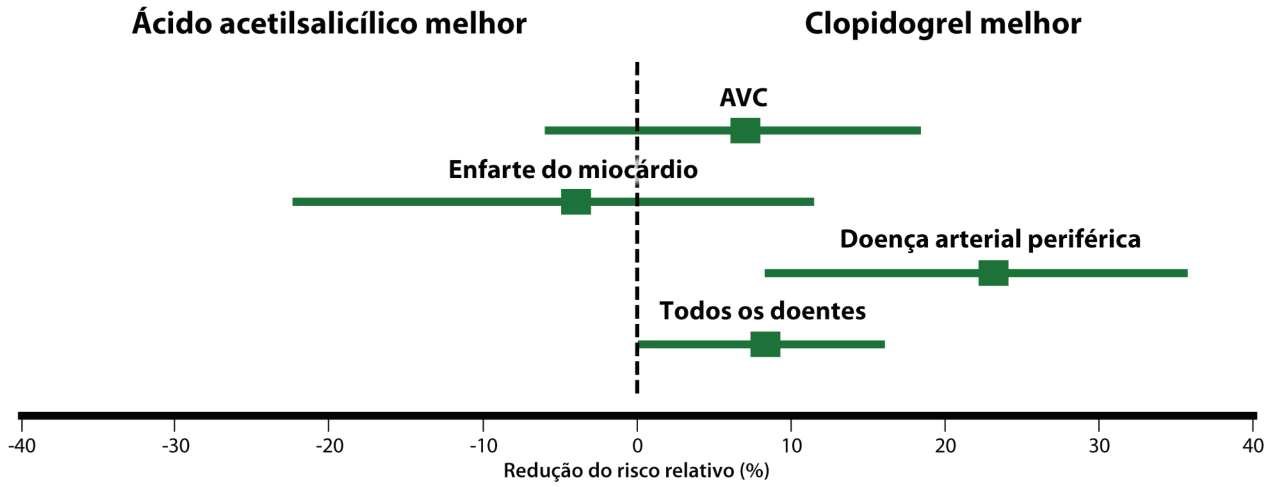

Quais os custos?

Custo mensal de tratamento para doses habituais de antiagregantes plaquetários.

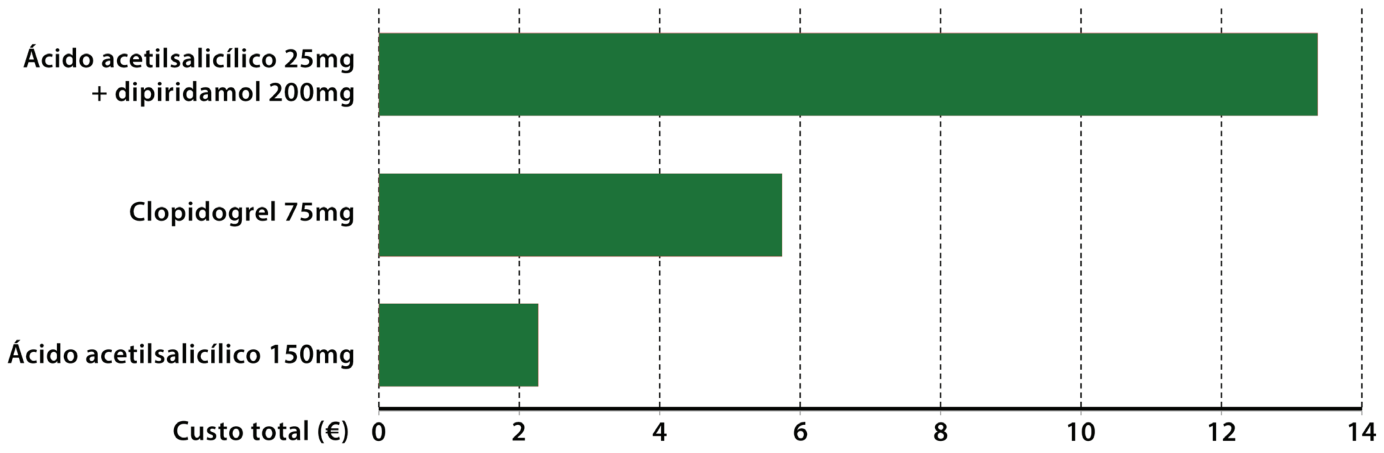

Fonte: Infarmed (1 de Maio de 2013)

Quando interromper para procedimentos invasivos?

Manter antiagregantes

- Endoscopia digestiva alta ou colonoscopia com ou sem biópsia
- Ecografia endoscópica sem biópsia
- Colangiopancreatografia retrógrada endoscópica sem esfincterectomia
- Tratamento dentário minor
- Cirurgia dermatológica
- Cirurgia oftalmológica cataratas

Interromper clopidogrel ou ácido acetilsalicílico + dipiridamol

- Polipectomia do cólon
- Ligação de varizes esofágicas
- Cirurgia cardíaca

Nos doentes de elevado risco cardiovascular, considerar fazer temporariamente ácido acetilsalicílico

Interromper todos os antiagregantes

- Ecografia endoscópica com biópsia
- Esfincterectomia endoscópica
- Cirurgia não cardíaca (manter ácido acetilsalicílico nas pessoas de risco cardiovascular elevado)

Adiar o procedimento

- Stent não medicado há menos de 6 semanas
- Stent medicado há menos de 6 meses o procedimento deve ser adiado

Se não for possível o adiamento, deve ser mantida a antiagregação dupla

Antiagregantes plaquetários

Indicação terapêutica	Tratamento recomendado
Síndroma coronária aguda	Clopidogrel + Ácido acetilsalicílico até 1 ano<br>Ácido acetilsalicílico depois
Intervenção coronária percutânea	Clopidogrel + Ácido acetilsalicílico até 1 ano<br>Ácido acetilsalicílico depois
Enfarte do miocárdio há +1 ano	Ácido acetilsalicílico<br>Clopidogrel pode ser alternativa em alguns doentes
Angina estável	Ácido acetilsalicílico
AVC isquémico / AIT	Ácido acetilsalicílico ou Clopidogrel*<br>ou Ácido acetilsalicílico + dipiridamol
Doença arterial periférica	Ácido acetilsalicílico ou Clopidogrel

* Sem indicação aprovada em pessoas com antecedentes de AIT.

Procedimentos invasivos e antiagregantes - o médico de família

Manter todos	Manter apenas ácido acetilsalicílico
Endoscopia digestiva alta ou colonoscopia (com ou sem biópsia)<br>Tratamento dentário minor<br>Cirurgia dermatológica<br>Cirurgia oftalmológica cataratas	Polipectomia do cólon<br>Cirurgia cardíaca<br>Cirurgia não cardíaca (parar ácido acetilsalicílico nas pessoas de baixo risco cardiovascular)

Pontos a reter

Não existe benefício em continuar por mais de um ano o clopidogrel após uma síndroma coronária aguda ou um cateterismo.

Manter o ácido acetilsalicílico indefinidamente nas pessoas com história de eventos coronários ou angina.

Nas pessoas com história de AVC não existe demonstração clara de benefício de qualquer dos antiagregantes.

O clopidogrel não possui indicação aprovada nas pessoas com antecendentes de AIT.

Na doença arterial periférica o clopidogrel poderá ter um benefício ligeiro sobre o ácido acetilsalicílico, mas qualquer das opções é válida.

Grupo de Informação Académica Independente
Faculdade de Ciências Médicas
Centro de Estudos de Doenças Crónicas (CEDOC)

Financiado por Ministério da Saúde / INSA, I.P
